# Influence of Season and Feedlot Location on Prevalence and Virulence Factors of Seven Serogroups of *Escherichia coli* in Feces of Western-Canadian Slaughter Cattle

**DOI:** 10.1371/journal.pone.0159866

**Published:** 2016-08-02

**Authors:** Kim Stanford, Roger P. Johnson, Trevor W. Alexander, Tim A. McAllister, Tim Reuter

**Affiliations:** 1 Alberta Agriculture and Forestry, Lethbridge, Alberta, Canada; 2 Public Health Agency of Canada, Guelph, Ontario, Canada; 3 Agriculture and Agri-Food Canada, Lethbridge, Alberta, Canada; USDA-ARS Eastern Regional Research Center, UNITED STATES

## Abstract

Pooled feces collected over two years from 1749 transport trailers hauling western-Canadian slaughter cattle were analysed by PCR for detection of *Escherichia* coli serogroups O26, O45, O103, O111, O121, O145, and O157. Sequential immunomagnetic separation was then used to collect bacterial isolates (n = 1035) from feces positive for target serogroups. Isolated bacteria were tested by PCR to confirm serogroup and the presence of *eae*, *ehxA*, *stx*_*1*_, and *stx*_*2*_ virulence genes. Based on PCR screening, serogroup prevalence in feces ranged from 7.0% (O145) to 94.4% (O103) with at least 3 serogroups present in 79.5% of samples. Origin of cattle affected serogroup PCR prevalence and O157 was most prevalent in feces from south-west Alberta (*P* < 0.001). All serogroups demonstrated seasonal variations in PCR prevalence, with O26, O45, O103, O121, and O157 least prevalent (*P* < 0.001) in cooler winter months, while uncommon serogroups O111 and O145 increased in prevalence during winter (*P* < 0.001). However, isolates collected during winter were predominantly from serogroups O103 and O45. No seasonal variation was noted in proportion of isolates which were Shiga toxin containing *E*. *coli* (STEC; *P* = 0.18) or positive for Shiga toxin and *eae* (enterohemorrhagic *E*. *coli*; EHEC; *P* = 0.29). Isolates of serogroups O111, O145, and O157 were more frequently EHEC than were others, although 37.6–54.3% of isolates from other serogroups were also EHEC. Shiga-toxin genes present also varied by geographic origin of cattle (*P* < 0.05) in all serogroups except O157. As cattle within feedlots are sourced from multiple regions, locational differences in serogroup prevalence and virulence genes imply existence of selection pressures for *E*. *coli* and their virulence in western-Canadian cattle. Factors which reduce carriage or expression of virulence genes, particularly in non-O157 serogroups, should be investigated.

## Introduction

Over 400 Shiga toxin-producing *Escherichia coli* (STEC) serotypes have been identified, but only a subset has been associated with severe human disease [1]. Serotype O157:H7 has been a food safety concern since the 1980’s [2], but incidence of disease due to non-O157 STEC has been increasing and now accounts for the majority of STEC infections in Ireland [3] and the USA [4]. Isolates of seven serogroups of *E*. *coli*: O26, O45, O103, O111, O121, O145 and O157 that carry Shiga toxin and intimin virulence genes have been classified as adulterants in beef in the USA [5].

The presence of virulence genes is important as serogroup alone does not accurately assess the ability of *E*. *coli* to cause human illness or the severity of disease [6]. Bacteriophage-encoded Shiga toxin 2 (*stx*_*2*_*)* is the principal virulence trait involved in hemolytic uremic syndrome [1], although other virulence factors such as Shiga toxin 1 (*stx*_*1*_), enterohemolysin (*exhA*) and intimin (*eae*) have been associated with STEC causing human disease [7, 8]. So far, at least 40 different virulence factors including subtypes of *stx*_*1*_ and *stx*_*2*_ have been identified. Defined combinations of virulence factors required for clinical infection have not been fully established, although progress has been made differentiating enterohemorrhagic *E*. *coli* (EHEC) from strains causing less-severe infections [9].

Cattle are a recognized reservoir of both O157 and non-O157 STEC [3, 10] although studies of the ecology of non-O157 STEC are limited due to a lack of reliable detection and characterization methods [11]. While methods for evaluation of O157 are well-established [12], study of non-O157 STEC is more challenging due to their diversity and inability of currently-available media to discriminate target non-O157 serogroups from generic *E*. *coli* [13, 14]. As efficacy of food safety systems could be improved with additional knowledge of non-O157 STEC ecology, the present study examined prevalence and virulence genes (*stx*_*1*_, *stx*_*2*_, *eae*, *ehxA*) of *E*. *coli* O26, O45, O103, O111, O121, O145, and O157 (Top 7 serogroups) and seasonality of their shedding in feces of western-Canadian cattle delivered for slaughter over a two-year period.

## Materials and Methods

### Sample collection

Sterilized scoops were used to collect fresh feces from floors within three compartments of transport trailers, immediately after delivery of 40 or more cattle to one of two federally-inspected slaughter facilities in Alberta, Canada. Permission was received for sample collection from both slaughter facilities. Feces from a trailer (5 scoops, ~ 400 g) were pooled within a Whirl-Pak bag and stored at 4°C in electric coolers until transport to the laboratory within 12 h. On each sampling day, a maximum of 46 pooled samples from trailers were collected, with collections occurring twice monthly (once for each slaughter plant) over 2 years. Drivers of trucks were surveyed as to feedlot and geographical origin of cattle (nearest town) within western Canada and at least 2 samples were collected per feedlot on each day of sampling.

### Initial screening for *E*. *coli* serogroup using PCR

Fecal samples were mixed by hand and a 15 g subsample was then mixed with 135 mL EC broth (EMD Millipore) using a Seward Model 400 stomacher (Cole-Palmer) at 230 rpm for 1 min. Fecal suspension (10 mL) was then transferred to a sterile culture tube and incubated for 6 h at 37°C. A 1 mL aliquot of the enriched culture was centrifuged at 8,000 X g for 10 min before extraction of DNA from the pellet using the NucleoSpin Tissue Kit (Machery-Nagel, Islington ON). Remaining enrichments were stored at 4°C for 18–24 h until used for immunomagnetic separation (IMS).

A multiplex PCR [11] detected the seven target *E*. *coli* serogroups. Briefly, PCR conditions included 50 nM O121; 40 nM O103, O111, O145, and O157; 25 nM O25 and O45 primers; 1 x QuantiFast master mix (Qiagen, Toronto ON), 2 μl DNA template and nuclease-free water in a final volume of 25 μL. Thermocycling conditions included an activation step of 95°C for 5 min followed by 45 cycles of 95°C for 45 s and 66°C for 60 s. Each PCR run contained positive (O26:H11, EC19960464; O45:H2, ED19940040; O103:H2, EC20010670; O111:NM, EC 20030053; O121:H19, EC20040083; O145:NM, EC2020231; O157:H7, strain R508), and negative (nuclease-free water) controls and was carried out on a Verti^™^ Dx Thermal Cycler (Applied Biosystems, Burlington ON).

### Isolation of bacteria by IMS and confirmation by PCR

Enrichments that were positive for one or more target serogroups were further analysed by IMS for each serogroup sequentially using RapidChek Confirm STEC Kits (Romer Labs Technology Inc, Union MO) according to manufacturer’s recommendations. Aliquots of bacteria-bead complex (50 μL) for each target serogroup were streak-plated with a cotton swab on MacConkey agar (MAC) and incubated at 37°C for 18–24 h. Three to 9 colonies per plate were selected and approximately half of each colony suspended in 40 μL 1 x TE buffer (10 mM Tris, 1 mM EDTA, pH 8.0). The suspension was heated to 95°C for 5 min and 2 μL used as DNA template for serogroup confirmation by PCR using conditions described above. The remainder of all colonies confirmed positive for a target serogroup by PCR were then removed from the plate, sub-cultured at 37°C overnight in tryptic soy broth (TSB) and stored in glycerol at -80°C. Once 25 PCR-confirmed isolates were collected per serogroup over at least 2 days of sampling in a season (spring, March-May; summer, June-August; fall, September-November; winter, December-February), IMS was not performed for that serogroup until the subsequent season.

### Detection of virulence genes

Glycerol stocks (1 mL) were grown overnight in 10 mL TSB at 37°C before extraction of DNA as described above for enrichments. Shiga toxin (*stx*_*1*_, *stx*_*2*_), intimin (*eae*) and enterohemolysin (*ehxA*) genes were detected using multiplex PCR, with the plasmid copy-number regulating gene (*repA*) used as an internal control [11]. Reaction mixtures contained 20 nM of each primer, 1 x QuantiFast master mix, nuclease-free water and 2 μL DNA template in 25 μL total reaction volume, and thermocycling conditions as previously described. Isolates were considered STEC if they contained either or both *stx*_*1*_ or *stx*_*2*_ genes and EHEC if they contained *eae* and either or both *stx* genes.

### Statistical Analyses

**Fig 1:**
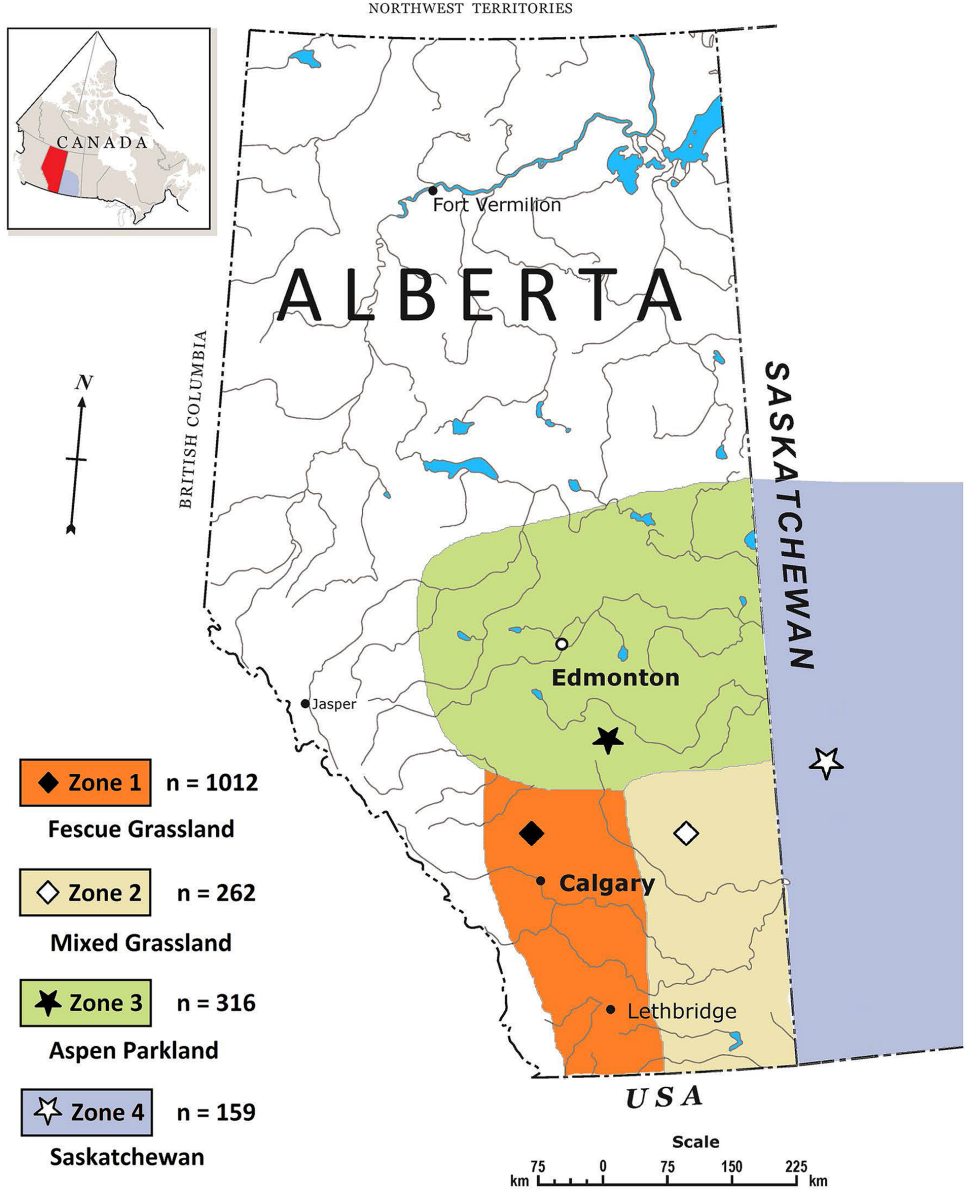
Zones of cattle origin and numbers of samples collected per zone. Zone 1 (n = 1012), fescue grassland; Zone 2 (n = 262), mixed grassland; Zone 3 (n = 316), aspen parkland; Zone 4 (n = 159), Saskatchewan. Reprinted from www.nrcan.gc.ca/earth-sciences/geography/atlas-canada/reference-maps/16846#provincial-and-territorial under a CC BY license, with permission from Natural Resources Canada, original copyright [2004].

Cattle originating from feedlots or auctions within the province of Alberta were grouped into 3 climactic zones, with the province of Saskatchewan considered a 4^th^ zone (Fig 1). Seasonal prevalence of serogroups detected by PCR and isolated by IMS was determined by generalized linear mixed models (Proc Glimmix, SAS 9.3, SAS Institute Inc, Cary, NC) using a binomial distribution. Model adjusted means (back-transformed to original scale) and 95% confidence intervals were reported, with transport trailer the experimental unit, season and origin of cattle considered as fixed effects and slaughter plant a random effect. *P* values < 0.05 were deemed significant.

## Results

### Origin of cattle and samples collected

During the 2-year period from May 2013 to June 2015, a total of 1761 transport trailers of western-Canadian slaughter cattle (40–45 animals) were sampled. Shipments which originated in Manitoba (n = 3), British Columbia (n = 4), and the Peace region of north-west Alberta (n = 5) were not included in statistical analyses as samples were not collected in all seasons. The transport trailer-loads included in analyses originated from 248 feedlots and 34 auction marts. The frequency that samples were collected from individual feedlots or auctions varied from high (samples collected monthly), to medium (10 to 20 samples collected per year), to low (less than 5 collected over two years). Geographic origin of the 1749 samples included in analyses and numbers of samples collected per zone are shown in Fig 1.

### Prevalence of serogroups and virulence genes of isolates

From PCR analyses after enrichment, serogroups fell into four distinct prevalence categories: rarely present (< 10% of samples; O111, O145), medium prevalence (> 50 and < 70% of samples; O121), high prevalence (> 75 and < 85% of samples; O26, O157), and extremely high prevalence (> 90% of samples; O45, O103; Table 1). Consequently, fecal samples positive for a single serogroup were rare (12/1749). The number of isolates collected from each serogroup after IMS followed similar trends to PCR prevalence, with the lowest number of O111 and O145 isolates (n = 19 and 40, respectively) and 200 or more isolates collected for O26, O45 and O103.

**Table 1:** Overall proportions of pooled fecal samples (n = 1749) positive for Top 7 serogroups by PCR, isolates collected after immunomagnetic separation (IMS) and virulence genes in isolates. Adjusted means are reported.

Percentage of isolates positive for virulence genes										
Serogroup	%PCR “+” (# PCR “+”)	% samples with isolates after IMS^z^ (# of assays)	Isolates/ serogroup^y^	*eae*	*ehxA*	*stx*_*1*_ only	*stx*_*2*_ only	*stx*_*1*_ and *stx*_*2*_	STEC^x^	EHEC^w^
**O26**	82.3^c^(1439)	15.0^d^(986)	210	74.5^c^	55.7^a^	52.2^d^^e^	3.4^a^	14.4^a^	69.7^a^^b^	54.3^b^
**O45**	93.1^d^(1628)	12.0^c^(1325)	200	44.2^a^	71.9^c^	35.6^b^	17.3^b^	27.6^b^	80.5^b^	37.6^a^
**O103**	94.4^d^(1651)	14.0^d^(1367)	245	63.1^b^	65.7^b^	42.7^b^^c^	10.0^b^	16.4^a^	69.1^a^^b^	42.6^a^
**O111**	8.2^a^(143)	0.6^a^(145)	19	97.5^d^	82.5^d^	64.7^e^	0.0^a^	35.2^b^	100.0^c^	98.5^d^
**O121**	66.1^b^(1156)	5.6^b^(1172)	138	49.1^a^	57.6^a^	45.6^c^^d^	10.3^b^	30.0^b^	85.9^b^	45.9^a^^b^
**O145**	7.0^a^(122)	1.2^a^(124)	40	97.5^d^	96.2^e^	64.3^e^	1.3^a^	11.9^a^	82.5^b^	79.1^c^
**O157**	78.8^c^(1378)	11.0^c^(1389)	183	87.2^d^	90.9^e^	9.7^a^	6.4^a^^b^	78.4^c^	94.5^c^	84.9^c^
**Total # analyses**	**1,749**	**6,508**	**1,035**	**1,035**	**1,035**	**1,035**	**1,035**	**1,035**	**1,035**	**1,035**

^a,b,c,d^Means within a column with different superscripts differ (*P* < 0.001).

^z^If 25 isolates were collected from a serogroup in a season over a minimum of 2 samplings, IMS was discontinued until the subsequent season.

^y^Three to 9 colonies per IMS plate were subdivided for PCR confirmation and isolates were saved from all PCR-confirmed colonies.

^x^STEC, having either or both s*tx*_*1*_ or *stx*_*2*_ genes.

^w^EHEC, having *eae* and either or both *stx*_*1*_ or *stx*_*2*_ genes.

Carriage of Shiga toxin genes varied markedly among serogroups. The majority of O26, O111 and O145 isolates were positive only for *stx*_*1*_, with few isolates of these serogroups positive only for *stx*_*2*_. In contrast, the O157 serogroup had a lower (*P* < 0.001) proportion of isolates positive only for s*tx*_*1*_ (9.7%) than all others. Highest proportions of STEC isolates were found in serogroups O111 and O157 (*P* < 0.001), but O157 isolates were more than twice as likely (*P* < 0.001) to possess both s*tx*_*1*_ and s*tx*_*2*_ than other serogroups. Intimin was most prevalent in O111, O145, and O157 (*P* < 0.001), whereas *ehxA* was most prevalent in O145 and O157 (*P* < 0.001). Accordingly, isolates of O111, O157, and O145 were more frequently classified as EHEC (*P* < 0.001) than O26, O45, O103, and O121 isolates. Less than 5% of isolates within each serogoup lacked all virulence genes.

### Origin of cattle and PCR positives for serogroup

Saskatchewan-origin feces had a lower prevalence of O103 and O157 (*P* < 0.001) than did feces from zones 1, 2 and 3 (Table 2). With the exception of zone 2, serogroup O121 was more prevalent (*P* < 0.001) in feces originating from zone 3 than those from other locations. In contrast, O111 was most prevalent in feces from zone 4 and zone 3, although zone 3 prevalence did not differ from that of other zones in Alberta. Serogroup O157 was most prevalent in cattle originating in zone 1 (*P* < 0.001).

**Table 2:** Influence of geographical origin of cattle on frequency of PCR positives for Top 7 serogroups in pooled fecal samples.

Percentage of PCR-positive samples by zone of cattle origin^z^								
Serogroup	Zone 1	95% CI	Zone 2	95% CI	Zone 3	95% CI	Zone 4	95% CI
O26	79.6^a^	74.2–84.1	81.7^a^	74.6–87.2	77.5^a^	70.3–83.3	75.6^a^	66.9–82.7
O45	92.9^b^	90.1–94.9	88.8^a^^b^	83.1–92.7	92.8^b^	88.7–95.6	87.4^a^	80.5–92.0
O103	93.7^b^	91.2–95.6	94.8^b^	90.8–97.1	95.5^b^	92.0–97.4	90.1^a^	84.0–92.1
O111	8.3^a^	6.0–11.3	8.8^a^	5.3–14.2	11.3^a^^b^	7.6–16.6	16.4^b^	10.8–24.2
O121	62.4^a^	52.3–68.9	66.8^a^^b^	58.0–74.6	74.2^b^	66.6–80.6	64.4^a^	54.6–73.0
O145	7.6^a^	5.4–10.5	8.4^a^	5.0–13.6	7.0^a^	4.3–11.2	5.9^a^	3.1–11.1
O157	85.2^c^	80.8–88.8	73.0^b^	64.7–80.0	77.2^b^	70.0–83.1	54.1^a^	44.2–63.6
**Total samples**	**1012**		**262**		**316**		**159**	

^ab^Means in a row with different superscripts differ (*P* < 0.001).

^z^Zones of cattle origin are illustrated in Fig 1.

### Origin of cattle and prevalence of STEC

Proportions of Shiga toxin genes in O157 isolates were consistent across locations (Table 3), but in non-O157 serogroups, Shiga-toxin genes varied with cattle origin. Isolates of O26 lacking *stx*_*1*_ and *stx*_*2*_ genes were most prevalent (*P* < 0.05) in zones 1 and 3, while O45 from Saskatchewan less often harbored only *stx*_*2*_ (*P* < 0.05) as compared to Alberta zones. Compared to zone 1, more (*P* < 0.05) 0103 isolates lacking both *stx*_*1*_ and *stx*_*2*_ were found in zone 2, although O111 isolates from zone 2 cattle were more likely to have both *stx*_*1*_ and *stx*_*2*_ than those from other locations.

**Table 3:** Prevalence of Shiga-toxin genes in isolates of the Top Seven serogroups according to zone of cattle origin and serogroup.

Percentage of PCR positives by zone of cattle origin					
Serogroup	*stx* genes	Zone 1	Zone 2	Zone 3	Zone 4
O26	*stx*_1_ only	46.6	60.7	57.9	56.7
	*stx*_2_ only	5.2	0	1.8	3.3
	*stx*_1_ & *stx*_2_	10.3	21.4	10.5	23.3
	no *stx*	37.9^b^	17.9^a^	29.8^b^	16.7^a^
**# isolates**		**95**	**28**	**57**	**30**
O45	*stx*_1_ only	33.1	36.1	37.5	50.0
	*stx*_2_ only	21.0^b^	16.7^b^	16.7^b^	0^a^
	*stx*_1_ & *stx*_2_	21.8	33.3	31.2	31.2
	no *stx*	24.2	13.9	14.6	18.8
**# isolates**		**100**	**36**	**48**	**16**
O103	*stx*_1_ only	42.1	37.2	45.1	44.0
	*stx*_2_ only	12.8	7.8	5.9	8.0
	*stx*_1_ & *stx*_2_	22.6^b^	5.9^a^	13.7^b^	16.0^b^
	no *stx*	22.6^a^	49.0^b^	35.3^a^^b^	32.0^a^^b^
**# isolates**		**118**	**51**	**51**	**25**
O111	*stx*_1_ only	75.0^b^	12.5^a^	76.9^b^	80.0^b^
	stx_2_ only	0	0	0	0
	*stx*_1_ & *stx*_2_	25.0^a^	87.5^b^	23.1^a^	20.0^a^
	no *stx*	0	0	0	0
**# isolates**		**3**	**4**	**4**	**8**
O121	*stx*_1_ only	33.3^a^	54.6^a^^b^	60.5^b^	34.4^a^
	*stx*_2_ only	14.3	0	9.3	12.5
	*stx*_1_ & *stx*_2_	27.0	36.4	25.6	37.5
	no *stx*	25.4	9.1	4.6	15.6
**# isolates**		**51**	**22**	**33**	**32**
O145	*stx*_1_	85.3^b^	63.2^a^^b^	28.6^a^	52.9^a^^b^
	*stx*_2_	6.0	5.3	0	0
	*stx*_1_ & *stx*_2_	3.0^a^	21.0^b^	51.1^c^	0^a^
	no *stx*	1.8^a^	10.5^a^	14.3^a^	47.1^b^
**# isolates**		**18**	**10**	**4**	**8**
O157	*stx*_1_ only	9.2	7.4	12.2	13.3
	*stx*_2_ only	7.7	11.1	2.4	0
	*stx*_1_ & *stx*_2_	77.1	74.1	80.5	80.0
	no *stx*	5.4	7.4	4.9	6.7
**# isolates**		**100**	**27**	**41**	**15**

^a,b,c^Means in a row with different superscripts differ (*P* < 0.05).

### Seasonality of serogroup detection by PCR

**Fig 2:**
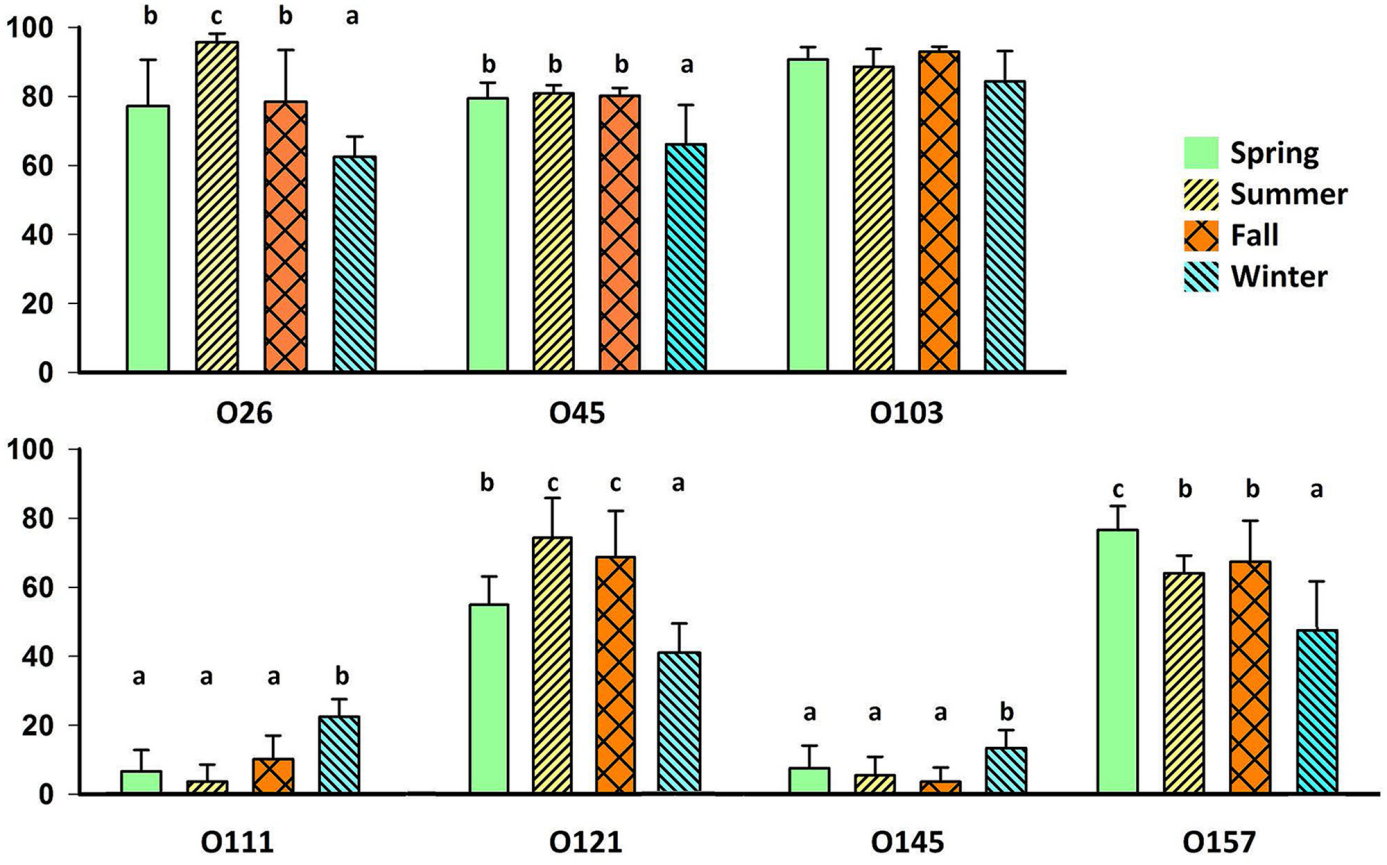
Seasonal^z^ PCR detection of Top Seven serogroups in feces of western Canadian slaughter cattle. Bars indicate 95% confidence intervals. Means within a serogroup with different superscripts differ (*P* < 0.001). ^z^Season: spring (March, April, May), summer (June, July, August), fall (September, October, November), winter (December, January, February).

Overall, serogroup detection by PCR was most likely to differ in the winter (December-February) as compared to other seasons (Fig 2). All serogroups showed seasonal variations, with the majority of serogroups (O26, O45, O103, O121, and O157) less prevalent (*P* < 0.001) in winter than in other seasons. In contrast, both O145 and O111 had a higher (*P* < 0.001) fecal prevalence in winter. The highest PCR prevalence of O26 and O45 occurred in summer, with O121 most prevalent in summer and fall seasons and O157 of highest PCR prevalence in spring (*P* < 0.001)

**Fig 3:**
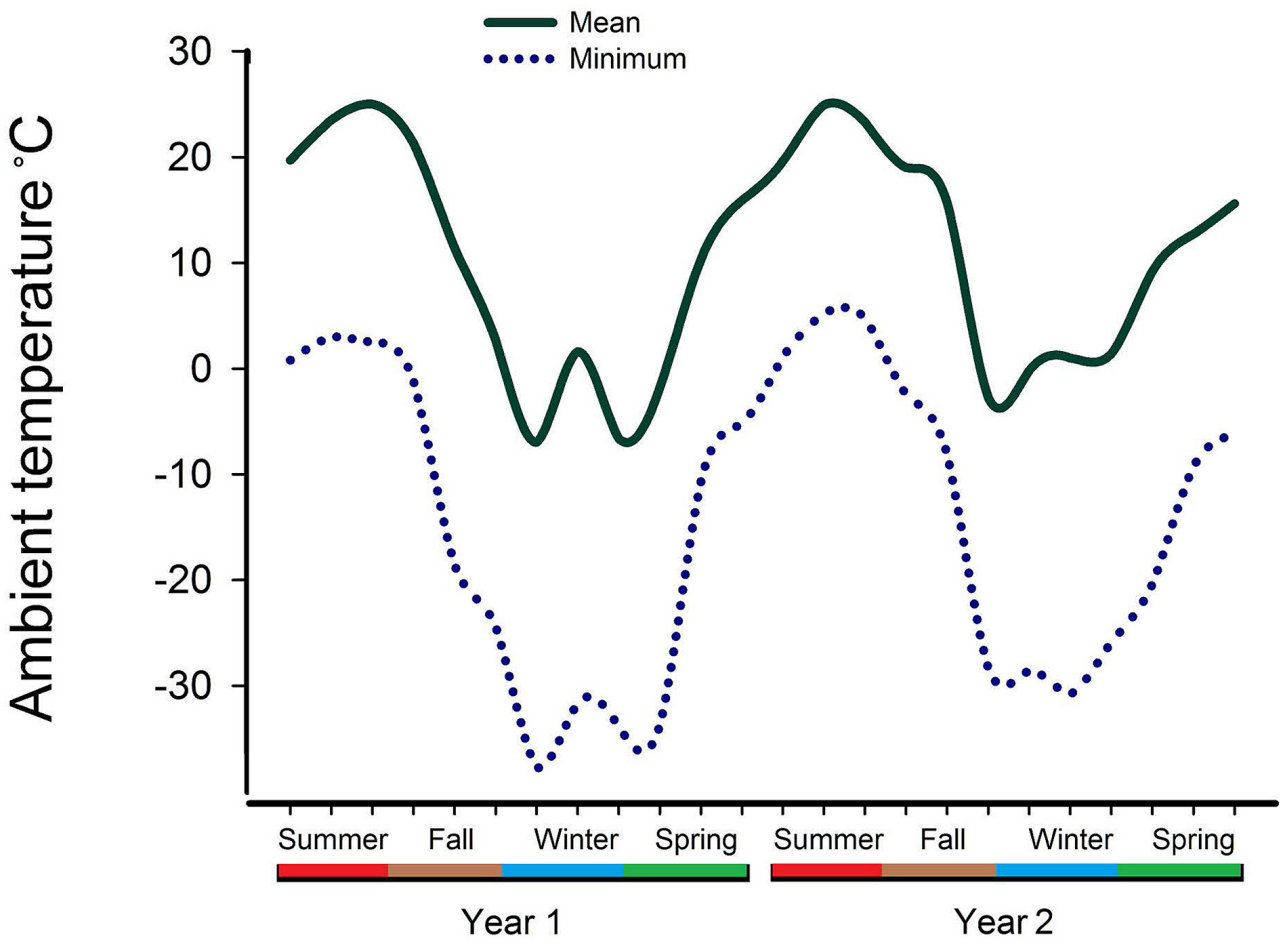
Mean and minimum temperatures (°C) for a location equidistant from the slaughter plants^z^ between June 1, 2013 and May 31, 2015. Ambient temperature (°C).

Seasonal temperatures were not consistent in both years of the study, with differences in severity of winters especially apparent (Fig 3). During the colder winter (year 1), PCR prevalence of serogroups O26, O45, O103, O121, and O157 was approximately half that of year 2 (*P* < 0.05; S1 Table. Mean PCR detection(%) of Top Seven by serogroup and season^z^ during each year of the study). Conversely, prevalence of serogroups O111 and O145 in the winter of year 1 was approximately twice that of year 2. Serogroups O45 and O103 maintained similar seasonal prevalence across both years in all seasons with the exception of winter, while other serogroups exhibited consistent prevalence across both years in one or two seasons and never in winter.

### Season of year and prevalence of serogroup isolates

Collecting 25 isolates per serogroup per season did not restrict numbers of O111, O145, and O121 isolates collected in any season and had minimal impact on numbers of O157 isolates collected (one sampling where IMS was not performed). As no serogroup reached maximum isolate number during winter, restricting isolate collection by season likely increased the proportion of isolates collected in winter for serogroups O26, O45 and O103, although there were more than twice as many samplings where IMS was not performed for O26 (n = 19) compared to O45 (n = 9) and O103 (n = 8; S2 Table. Numbers of isolates collected per month of study).

**Fig 4:**
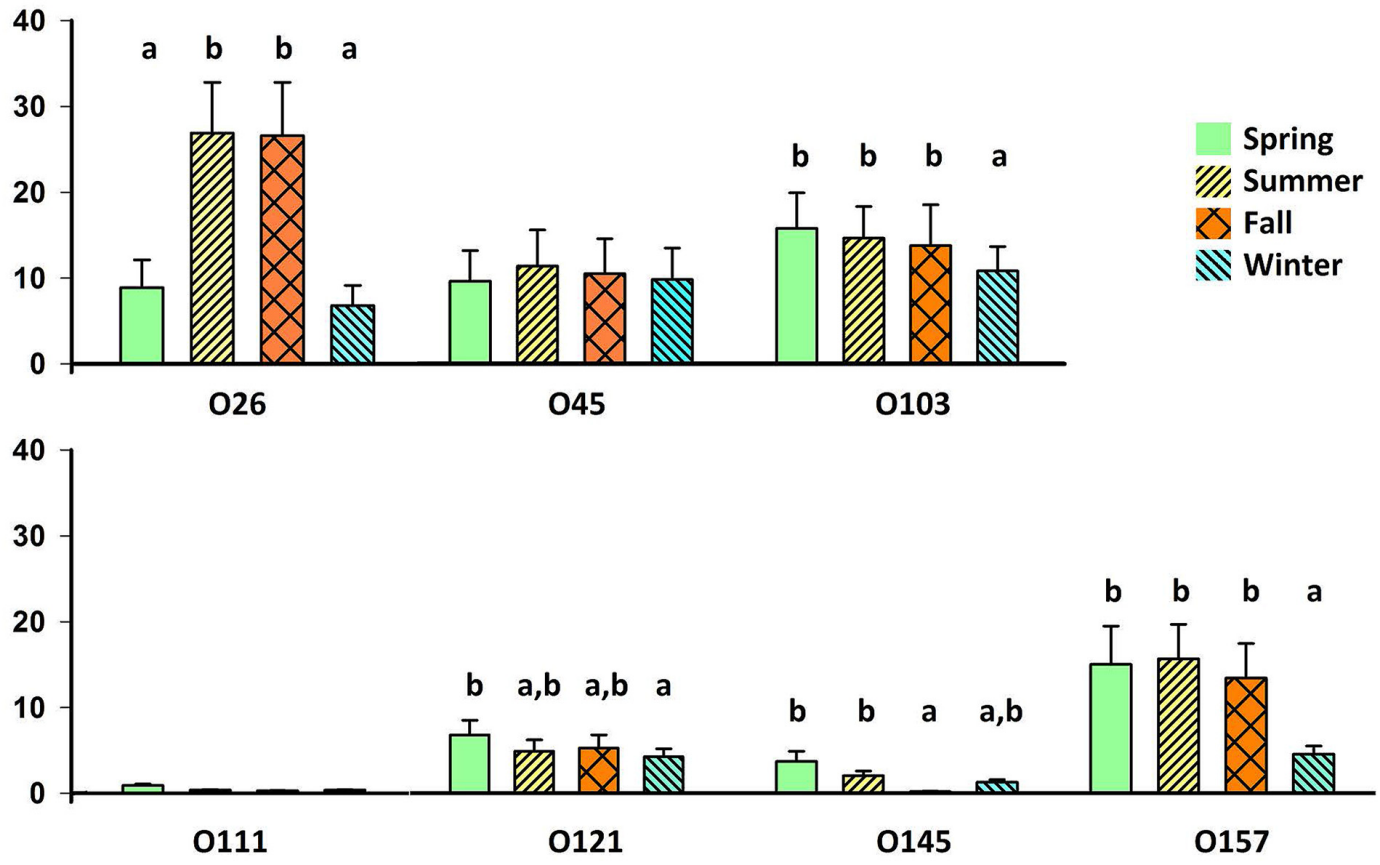
Seasonal^z^ proportions of fecal samples yielding isolates of Top 7 serogroups^y^. Bars indicate 95% confidence intervals. Means within a serogroup with different superscripts, differ (*P* < 0.001). ^z^Season: spring (March, April, May), summer (June, July, August), fall (September, October, November), winter (December, January, February). ^y^If 25 isolates were collected from a serogroup over a minimum of 2 samplings in a season, IMS was discontinued for subsequent samplings until the following season.

For serogroup O45, equal proportions of samples yielded bacterial isolates across all seasons (Fig 4). In other serogroups, collection of isolates appeared to be strongly seasonal. During winter, collection of O157 isolates was approximately 3 times less likely (*P* < 0.001) compared to other seasons, while similar trends were observed for O26 in the spring and winter as compared to summer and fall. In contrast, collection of O145 isolates was more likely in the spring, than in the fall (*P* < 0.001). Similar to O145, O111 isolates tended to be most likely collected in the spring, although too few O111 isolates were collected to confirm seasonal patterns. Collection of O103 isolates was less likely in winter than in other seasons (*P* < 0.001), although seasonal changes were modest for this serogroup compared to O26 and O157. No differences were found in seasonal proportions of STEC (*P =* 0.18) or EHEC (*P* = 0.29) among isolates of Top 7 serogroups.

### Seasonal combinations of serogroups detected by PCR

As serogroups varied in fecal prevalence seasonally, certain combinations of serogroups detected by PCR were also indicative of seasons (Table 4). The most frequently-observed combination (pattern I, 666/1749 samples) was positive for all serogroups except for O111 and O145 and was most prevalent in summer (*P* < 0.05). The second most-frequently observed combination had heightened prevalence during spring (pattern II; *P* < 0.05) and was positive for all serogroups except O111, O121 and O145. A combination least common in summer (pattern IV; *P* < 0.05) lacked O26, O111 and O145 in contrast to a combination most common in winter and spring (pattern V; *P* < 0.05) that was positive only for O45, O103 and O157. Some common combinations of PCR positives (patterns VI and VIII) remained at similar prevalence year-round.

**Table 4:** Seasonal prevalence (%) of the most frequent combinations of Top 7 serogroups detected by PCR from pooled samples of cattle feces collected at delivery to slaughter (n = 1,749).

Serogroup PCR detection								Season^z^				Totals
Pattern^y^	O26	O45	O103	O111	O121	O145	O157	Spring	Summer	Fall	Winter	# samples (% samples)
I	1	1	1	0	1	0	1	34.0^b^	49.2^c^	39.5^b^	15.3^a^	**666(38.1)**
II	1	1	1	0	0	0	1	20.2^c^	7.2^a^	11.2^b^	11.8^b^	**234(13.4)**
III	1	1	1	0	1	0	0	4.0^a^^b^	16.2^c^	6.1^b^	2.1^a^	**131(7.5)**
IV	0	1	1	0	1	0	1	7.2^b^	1.6^a^	7.1^b^	6.3^b^	**99(5.7)**
V	0	1	1	0	0	0	1	6.8^c^	0.1^a^	2.4^b^	10.2^c^	**87(5.0)**
VI	1	1	1	0	0	0	0	3.6	4.4	1.9	1.5	**65(3.7)**
VII	1	1	1	1	1	0	1	2.4^a^^b^	1.2^a^	4.7^b^^c^	5.3^c^	**61(3.5)**
VIII	1	1	1	0	1	1	1	2.8	2.3	2.4	2.3	**47(2.7)**
**Total**												**1390(79.5)**

^a,b,c^Means in a row with different superscripts differ (*P* < 0.001).

^z^Season: spring (March, April, May), summer (June, July, August), fall (September, October, November), winter (December, January, February).

^y^Pattern: 1 = PCR positive, 0 = PCR negative.

## Discussion

### Prevalence and virulence genes of serogroups

In the present study, overall and relative prevalence of Top 7 serogroups determined by PCR screening of feces was similar to that reported for O26, O103, O111, O121, and O157 from feedlot cattle feces in the USA [14], although those authors did not detect O145. Dargatz et al.[15] reported similar relative prevalence to the present study among Top 7 serogroups in feces collected from a survey of USA feedlots, although overall fecal prevalence was substantially higher for all serogroups evaluated in the present study. Similarly, Cernicchiaro et al. [16] reported that 33% of 960 fecal samples from feedlot cattle were PCR positive for two or more serogroups, compared to more than 79% of 1,749 samples in the present study (Table 4). Collecting pooled fecal samples from the floors of transport trailers instead of individual cattle [8, 16] and from cattle originating from feedlots and auction marts across western Canada instead of a single feedlot in the USA [17] while also taking advantage of improvements in detection methods from earlier studies [8, 15] likely contributed to the high PCR prevalence of Top 7 serogroups noted in the present study. Due to integration of the North American beef industry and shipment of cattle in both directions across the US border [18], a similar relative prevalence of Top 7 serogroups in Canada and the USA would be expected.

As with previous studies [8, 17], O111 and O145 were less frequently detected in feces than were other serogroups evaluated. However, the low frequency of collection of isolates from these two serogroups may also indicate a lack of selectivity in the IMS kits used. Similarly, Bai et al. [19] and Cernicchiaro et al. [16] found IMS beads for O111 often yielded colonies of O103 due to non-specific binding of antibodies, while Cooley et al. [20] concluded that using specific O-type IMS kits did not significantly improve isolation of the target serogroup. Isolates of O111 obtained in the present study were similar to environmental and outbreak strains of this serogroup surveyed by Diodati et al. [21] in that all harbored *stx*_*1*_ and only a subset carried *stx*_*2*_. That all O111 colonies isolated were STEC was unexpected, but perhaps reflects the stability of the cryptic prophage carrying *stx*_*1*_ associated with this serogroup [22]. Alternatively, as O111 has been linked to 19% of STEC infections in the USA [4] even though rates of detection and/or isolation of O111 in previous studies have been low [15, 16, 17, 23], heightened virulence of O111 isolates is implied.

For all serogroups, more than 50% of isolates collected in the present study were STEC, and > 75% of O111, O145 and O157 isolates were also EHEC. A high proportion of O157 EHEC would be expected [17, 24], although O157 isolates lacking virulence genes have also been reported from feedlot cattle feces [12]. Previous studies collected only STEC isolates [20, 25, 26], screened for the Top 7 serogroups using PCR only [15], or collected few isolates [23, 27], making comparisons among studies difficult. However, proportions of STEC and EHEC isolates of non-O157 *E*. *coli* have generally been lower in previous studies than presently reported.

In a survey of Scottish cattle, Pearce et al. [8] found 2/168 O103 and 2/43 O145 isolates possessed *stx* genes. Thomas et al. [24] found a similarly low prevalence of O103, O26, and O145 STEC on hides of slaughter Irish cattle, although numbers of isolates collected per serogroup were < 25 with the exception of O103 where 109 isolates were evaluated. Dewsbury et al. [17] found no STEC isolates of O45 or O121, while 10/340 O103 isolates were EHEC, although similar to the present study, O145 was most likely to be EHEC (6/18 isolates). Serogroups O145 and O157 are thought to be closely related, sharing core virulence determinants before evolving into separate sub-lineages [28], possibly explaining the heightened virulence of O145 relative to other non-O157 *E*. *coli*. Serogroup O111 is thought to be most closely linked to O26, although evolution of *E*. *coli* is complex, with H antigens often more informative of evolutionary history than O serogroups [29]. Accordingly, comparison of H antigens associated with O111 and O26 isolates collected in the present study might explain differences in virulence determinants noted for these serogroups, although serotyping of collected isolates remains in progress.

Even among studies with similar data sets, a direct comparison of serogroup and virulence gene prevalence is difficult. Cooley et al. [20] found significant media bias in detection of both STEC serogroup and virulence gene profiles. We used EC broth without antimicrobials as it has been recommended for enrichment of non-O157 *E*. *coli* in feces to avoid underestimation of STEC prevalence [30]. Cernicchario et al. [16] used similar media but used a PCR assay [19] with reduced sensitivity for detection of O serogroups compared to that of the present study [11]. As *stx* genes are resident on prophages, the use of antimicrobials in media may convert lysogenic to lytic bacteriophages [31], resulting in potential loss of a fraction of *stx* genes from isolates, as *stx* genes are present on both inducible and cryptic (non-inducible) bacteriophages [32].

Although O157:H7 has been well characterized [12, 33], fewer non-O157 *E*. *coli* have been isolated and evaluated. Compared to O157, non-O157 *E*. *coli* are highly diverse [20] and produce heterogeneous clinical outcomes [29]. Even when isolates have been fully serotyped, virulence gene profiles have been reported to be highly variable in non-O157 *E*. *coli* of the same serotype [25]. The diversity of *stx* genotypes present in non-O157 *E*. *coli* could possibly be due to diverse populations of Shiga toxin-encoding bacteriophages as suggested by Carter et al. [34] in a survey of O145 isolated from cattle, wildlife, and the environment.

Virulence gene profiles of isolates collected in the present study demonstrated that all serogroups have the potential to cause human disease, with O111, O145 and O157 isolates possibly of greatest disease risk due to a higher proportion of EHEC [35]. However, expression of *E*. *coli* virulence genes is variable [34, 36] and further studies will be necessary to clarify relationships between virulence genes and human disease.

### Effects of origin of cattle on serogroup prevalence and virulence genes

Previous studies [15, 37] have not reported locational differences in frequency of detection of STEC serogroups among feedlots in the USA, likely as limited feedlots were sampled due to travel and sample-processing logistics. However, regionally endemic strains of O157:H7 are known to exist in the UK [38] and farm-specific STEC serotypes have been found in Irish cattle [25]. As western-Canadian beef processing is largely confined to the two abattoirs surveyed in this study, cattle were sampled from a total of 248 feedlots and 34 auction marts, including all of the estimated 150 feedlots in Alberta and Saskatchewan with capacities of 1000 head or larger, 98 feedlots with < 1000 head capacity, and 65% of the livestock auctions in these provinces [39].

In the present study, regional variations in serogroup prevalence were most marked between Saskatchewan- and Alberta-origin feces (Table 2), with samples from Saskatchewan less often positive for O103 and O157 and more often positive for O111 than Alberta samples. Similarly, regional variations in serogroup prevalence were found in a survey of 338 farms, as O145 was not detected on any farms in north-east Scotland [8]. Pastured cattle have shown a preponderance of O121 compared to other non-O157 STEC [40], although feedlot cattle from the regions of Alberta with highest prevalence of O121 in the present study would have likely received high-grain diets. Reasons for locational differences in prevalence of Top 7 serogroups are unclear as calves present in many feedlots would have been sourced from across western Canada. Consequently, selection pressures for or against specific serogroups likely exist within feedlots. For example, only 2 Saskatchewan feedlots have capacities of 10,000 head, whereas the majority of Alberta feedlots would be of this scale or larger [39]. The fact that zone 1 had the highest prevalence of O157 is interesting as this region has the highest density of feedlot cattle in Canada [41] and has also been reported to be at increased risk for human cases of infection due to O157:H7 [42].

Locational differences in virulence genes of isolates were confined to non-O157 STEC, further demonstrating the heterogeneity of these serogroups compared to O157. Locational differences in virulence genes would support the existence of selection pressures for virulence traits among EHEC in agricultural environments [34], although these are as yet poorly-defined. Similarly, a highly-virulent strain of O157:H7 has become increasingly common in UK cattle in the past 25 years, but reasons for its rise to dominance are unknown [38].

While *E*. *coli* may become increasingly virulent by incorporation of *stx*-encoding prophages [32], O26 EHEC has been shown to rapidly lose *stx* genes and virulence in human patients [43]. It has been proposed that the loss of *stx*_*2*_ genes or their inactivation may contribute to long-term fitness and maintenance of O157:H7 within the gastrointestinal tracts of cattle [36]. As both presence and expression of virulence genes appear rapidly mutable, geographical differences in virulence among Top 7 serogroups in cattle feces are likely continuously changing and related to selection pressures such as diet, antimicrobial use, climate, animal density, or other stressors.

### Seasonality of shedding

Combinations of serogroups in feces present in only 1 or 2 samples were largely confined to winter, possibly due to increased intensity of selection pressures such as ambient temperature. Climactic variability increases the difficulty in determining seasonal prevalence of *E*. *coli* shedding, while collecting samples from individual animals instead of pooling feces likely also explains some contradictions observed among previous studies. However, collecting two years of data gives credence to overall trends observed in the present study as previous studies typically evaluated seasonal fecal prevalence of non-O157 *E*. *coli* within a single year [8, 17, 44].

Similar to the present study, O103 was found to be the most prevalent serogroup in winter [17], although in contrast to the present study those authors found O157 to be most prevalent during the summer. In the present study, O103 declined significantly in prevalence only in winter, although Pearce et al. [8] found less O103 in feces during the spring compared to the summer and fall. Dewsbury et al. [17] found O45 to be second most common serogroup shed in winter, similar to results of the present study. Ennis et al. [25] did not find seasonal peaks in STEC shedding, but 33 serotypes were detected, the majority of which were not Top 7 serogroups. Similarly, Ekiri et al. [45] did not find seasonal variation in shedding of Top 7 serogroups, although a small population of 48 cows and calves was sampled. Barocky-Gallager et al. [44] found more non-O157 STEC in fecal samples in the spring and fall than in the summer and winter, but did not identify individual serogroups. Pearce et al. [8] found more O26 in feces during the summer than spring but did not detect seasonal variation in shedding of O145 due to a limited number of positive samples. Contrary to the present study where seasonal variation was not found in prevalence of virulence genes in isolates of Top 7 serogroups, Dewsbury et al. [17] found no virulence genes in isolates of O26, O45, O103, or O121 collected in winter, possibly as these authors evaluated a single feedlot which may have had a feedlot-specific low prevalence of virulence genes.

Seasonality of shedding of O111 and O145 has not been previously addressed as few positive fecal samples have previously been detected [3, 8, 17, 45]. Results of the present study demonstrate increasing prevalence of O111 and O145 in winter when fecal prevalence of other serogroups was declining. A reduction in shedding of O157 in feces during colder months has been noted [46, 47] and dynamic shifts in microbial populations of cattle feces have been established [48]. Seasonal shifts in combinations of serogroups present in feces were also observed (Table 4), with many of these differences traceable to changes in O111 and O145 prevalence relative to other serogroups.

### Seasonality of survival of isolates of Top 7 serogroups

As fecal samples were collected from the floors of transport trailers, seasonal trends noted for collection of isolates after IMS may reflect survival of isolates from Top 7 serogroups in the environment. For some serogroups (O26, O103, O121, O157), the likelihood of isolate collection corresponded to seasonal detection by PCR. Although serogroup O45 had reduced fecal prevalence during the winter, the proportion of isolates collected in all seasons was similar, perhaps reflecting a heightened cold tolerance. Although both O111 and O145 serogroups had elevated winter prevalence in feces as determined by PCR, the likelihood of collecting isolates was not similarly improved in winter, perhaps indicating limited cold tolerance, or more non-culturable isolates in winter. However, as relatively few isolates of O111 and O145 were collected, environmental survival of these and other serogroups requires additional study. A recent study [49] has speculated that serogroup-specific differences in survival may be responsible for human infections due to O26 peaking approximately 2 months earlier than those due to O157.

Heightened survival of O157:H7 in the environment is known to occur in warmer months, along with a seasonal peak in human disease [47]. In contrast, human infections collectively associated with non-O157 *E*. *coli* appear to exhibit less seasonality [50]. The lack of seasonal variation in virulence genes carried by isolates, year-round shedding and survival of the prevalent O103 serogroup, and potentially heightened cold-season survival of serogroups such as O45 observed in the present study may also account for the reduced seasonality of non-O157 infections in humans.

### Comparison of O157 to other serogroups

Although similar proportions of O111, O145 and O157 were EHEC when isolated, both O111 and O145 were rare, while O157 was present in 78.8% of feces. Shiga-toxin genes carried by non-O157 serogroups varied by location and were generally less prevalent compared to those of O157 which did not vary in prevalence across western Canada. However, O157 was strongly seasonal and a low proportion of O157 were detected and/or isolated in the winter compared to serogroups O103 and O45. Excluding O111 and O145, between 58.9–80.5% of non-O157 *E*. *coli* were STEC, and 37.6–54.3% were EHEC. Human disease due to O157 has been declining relative to that caused by non-O157 STEC [3], which can partly be attributed to improved isolation of non-O157 *E*. *coli*. Results of this study imply that collectively, serogroups O26, O45, O103, O111, O121, and O145 should exceed the human health risk of O157 due to heightened year-round fecal prevalence and simultaneous presence of multiple EHEC serogroups in cattle feces. However, human infection with O157 in Canada exceeds that due to non-O157 *E*. *coli* [51].

## Conclusions

Multiple Top 7 serogroups were common in cattle feces, with at least 3 serogroups present in 79.5% of samples and individual serogroups such as O45 and O103 present in > 90% of samples. Prevalence of serogroups and Shiga toxin profiles of non-O157 *E*. *coli* isolates varied by geographical location of feedlot, although cattle for these feedlots would have been sourced from across western Canada, implying existence of regional selection pressures for serogroup and carriage of Shiga-toxin genes. All serogroups exhibited seasonal variation in prevalence, but in contrast to others, prevalence of O111 and O145 increased during winter. Proportion of O45 isolates collected was similar during all seasons, implying increased survival during winter.

Serogroups O111, O145 and O157 had highest proportions of EHEC isolates, with lowest proportions of EHEC from O45, O103 and O121 serogroups. Even though non-O157 EHEC isolates collected during the two years of this study outnumbered O157 EHEC by approximately 3:1, human infections in Canada from O157 exceed those due to non-O157 serogroups [51]. Differences between prevalence and human infection among O157 and non-O157 *E*. *coli* may be linked to increased ease of isolation of O157, but may also be due to more-variable pathogenicity of non-O157 *E*. *coli*. In the present study, virulence genes carried by O157 were consistent in all regions sampled, while locational variations were present in the virulence genes carried by non-O157 *E*. *coli*. Although selection pressures exist to enhance pathogenicity of *E*. *coli* [34], fitness of *E*. *coli* in cattle can be increased by gene loss or loss of functional Shiga-toxin genes [36] and selection pressures which reduce carriage or expression of virulence genes, particularly by non-O157 serogroups, should be investigated.

## Supporting Information

S1 Table Mean PCR detection (%) of Top Seven by serogroup and season^z^ during each year of the study. (DOCX)

S2 Table Numbers of isolates collected per month of study. For serogroups O26, O45, O103 and O157 number of IMS assays performed per month is in brackets. For other serogroups 2 IMS assays were performed per month.
(DOCX)
