# Supplementary material for: Influence of Season and Feedlot Location on Prevalence and Virulence Factors of Seven Serogroups of Escherichia coli in Feces of Western-Canadian Slaughter Cattle
Source: PLoS One. 2016 Aug 2;11(8):e0159866. doi: 10.1371/journal.pone.0159866 (PMC4970752; doi:10.1371/journal.pone.0159866)
Supplement: S1 Table — (DOCX) [file pone.0159866.s001.docx]

**S1 Table**. **Mean PCR detection (%) of Top Seven by serogroup and season^z^ during each year of the study.**

**Spring Summer Fall Winter**

| Serogroup | Year 1 | Year 2 | Year 1 | Year 2 | Year 1 | Year 2 | Year 1 | Year 2 |
| --- | --- | --- | --- | --- | --- | --- | --- | --- |
| O26 | 76.0^b^ | 78.8^b^ | 92.7^c^ | 98.0^c^ | 71.7^b^ | 86.2^c^ | 37.5^a^ | 65.9^b^ |
| O45 | 93.3^c^ | 95.5^c^ | 92.7^c^ | 98.8^c^ | 92.5^c^ | 98.6^c^ | 42.5^a^ | 80.9^b^ |
| O103 | 93.8^b^ | 96.3^b^ | 88.8^b^ | 99.7^b^ | 96.7^b^ | 98.6^b^ | 57.5^a^ | 92.3^b^ |
| O111 | 2.2^a^ | 9.8^a^ | 4.7^a^ | 12.4^ab^ | 5.4^a^ | 14.8^b^ | 45.0^c^ | 18.8^b^ |
| O121 | 55.6^b^ | 62.9^bc^ | 74.3^c^ | 85.4^d^ | 70.4^c^ | 79.5^c^ | 12.5^a^ | 48.5^b^ |
| O145 | 4.4^a^ | 11.0^b^ | 8.9^ab^ | 3.2^a^ | 2.3^a^ | 4.8^a^ | 22.5^c^ | 12.2^b^ |
| O157 | 87.6^c^ | 89.8^c^ | 85.1^c^ | 95.6^c^ | 65.8^b^ | 92.4^c^ | 32.5^a^ | 80.1^c^ |

^a,b,c,d^ Means in a row with different superscripts differ (*P* < 0.05).

^z^Season: spring(March, April, May), summer (June, July, August), fall (September, October, November), winter (December, January, February).
