# Supplementary material for: Influence of Season and Feedlot Location on Prevalence and Virulence Factors of Seven Serogroups of Escherichia coli in Feces of Western-Canadian Slaughter Cattle
Source: PLoS One. 2016 Aug 2;11(8):e0159866. doi: 10.1371/journal.pone.0159866 (PMC4970752; doi:10.1371/journal.pone.0159866)
Supplement: S2 Table — For serogroups O26, O45, O103 and O157 number of IMS assays performed per month is in brackets. For other serogroups 2 IMS assays were performed per month. (DOCX) [file pone.0159866.s002.docx]

**S2 Table. Numbers of isolates collected per month of study. For serogroups O26, O45, O103 and O157 number of IMS assays performed per month is in brackets. For other serogroups 2 IMS assays were performed per month.**

**Serogroup**

| Year | Month | O26 | O45 | O103 | O111 | O121 | O145 | O157 |
| --- | --- | --- | --- | --- | --- | --- | --- | --- |
| 2013 | Jun | 35(2) | 6(2) | 1(2) | 0 | 3 | 1 | 5(2) |
| 2013 | Jul | 0(0) | 3(2) | 2(2) | 2 | 2 | 0 | 7(2) |
| 2013 | Aug | 0(0) | 5(2) | 25(1) | 0 | 12 | 1 | 30(2) |
| 2013 | Sep | 32(2) | 9(2) | 13(2) | 3 | 14 | 1 | 13(2) |
| 2013 | Oct | 0/0 | 12(2) | 20(1) | 3 | 4 | 0 | 11(2) |
| 2013 | Nov | 0/0 | 0(2) | 0(2) | 0 | 3 | 0 | 1(2) |
| 2013 | Dec | 0(2) | 3(2) | 1(2) | 0 | 2 | 0 | 0(2) |
| 2014 | Jan | 5(2) | 8(2) | 8(2) | 0 | 2 | 1 | 2(2) |
| 2014 | Feb | 3(2) | 4(2) | 14(2) | 0 | 4 | 0 | 1(2) |
| 2014 | Mar | 0(2) | 16(2) | 18(2) | 1 | 9 | 2 | 3(2) |
| 2014 | Apr | 7(2) | 12(2) | 20(1) | 0 | 14 | 5 | 11(2) |
| 2014 | May | 0(2) | 0(0) | 0(0) | 1 | 3 | 3 | 13(2) |
| 2014 | Jun | 28(2) | 14(2) | 8(2) | 1 | 8 | 8 | 9(2) |
| 2014 | Jul | 0(0) | 11(2) | 15(2) | 0 | 3 | 5 | 10(2) |
| 2014 | Aug | 0(0) | 0(0) | 17(1) | 0 | 5 | 0 | 1(1) |
| 2014 | Sep | 32(2) | 16(2) | 18(2) | 0 | 5 | 2 | 8(2) |
| 2014 | Oct | 0(0) | 6(2) | 10(1) | 0 | 2 | 0 | 5(2) |
| 2014 | Nov | 0(0) | 18(1) | 0(0) | 0 | 2 | 0 | 5(2) |
| 2014 | Dec | 13(2) | 4(2) | 10(2) | 0 | 5 | 0 | 3(2) |
| 2015 | Jan | 7(2) | 17(2) | 12(2) | 3 | 2 | 1 | 4(2) |
| 2015 | Feb | 5(1) | 6(2) | 7(1) | 0 | 12 | 6 | 8(2) |
| 2015 | Mar | 10(2) | 10(2) | 9(2) | 4 | 5 | 2 | 8(2) |
| 2015 | Apr | 33(2) | 20(2) | 4(2) | 0 | 5 | 2 | 13(2) |
| 2015 | May | 0(0) | 0(0) | 13(2) | 1 | 12 | 0 | 12(2) |
| Total |  | 210 | 200 | 245 | 19 | 138 | 40 | 183 |
